# Recurrent lung nodules as a presentation of ventricular septal defect‐related endocarditis

**DOI:** 10.1002/rcr2.446

**Published:** 2019-06-01

**Authors:** Adam Trytell, Jonathan Darby, Matthew Conron, Andrew Newcomb, Andrew Burns

**Affiliations:** ^1^ Department of Respiratory Medicine, Infectious Diseases, Cardiothoracic Surgery and Cardiology St. Vincent's Hospital Melbourne Melbourne Australia

**Keywords:** Infective endocarditis, inflammation, lung nodules, *Streptococcus mutans*, ventricular septal defect

## Abstract

Infective endocarditis is an uncommon microbial infection of the endocardial surface of the heart. Patients with structural heart disease, such as a ventricular septal defect, are at higher risk for infective endocarditis and clinicians must have a high index of suspicion in such patients presenting with recurrent fevers. We present a patient with a known ventricular septal defect presenting with recurrent fevers associated with migratory lung nodules following a “low‐risk” dental procedure without antibiotic prophylaxis. The unusual presentation delayed the diagnosis of the migratory lung lesions as septic pulmonary emboli and consequentially the diagnosis of ventricular septal defect related infective endocarditis. The patient made an uneventful recovery following antibiotic therapy and surgical intervention.

## Introduction

Infective endocarditis (IE) is an uncommon microbial infection of the endocardial surface of the heart 1. Patients with structural heart disease, such as a ventricular septal defect (VSD), are at higher risk for IE 2. We present a patient with a known VSD, who repeatedly presented with an usual presentation of recurrent and migratory lung nodules, which were later diagnosed as septic pulmonary emboli, likely secondary to IE that was further complicated by possible vertebral osteomyelitis on the background of a dental procedure without antibiotic prophylaxis.

## Case Report

A 64‐year‐old male with a known VSD, who had not experienced any previous VSD‐related complications, underwent a dental crown implantation without antibiotic prophylaxis in September 2016. Three months later he experienced fevers, sweats, and a dry cough, which spontaneously resolved after several weeks without antibiotic treatment. His symptoms recurred in June 2017 and at this time were associated with raised inflammatory markers. Further investigation, including a computed tomography (CT) chest, identified multiple peripheral lung lesions that were initially presumed to be malignant. Once again, his symptoms spontaneously resolved without any antibiotics. A follow‐up CT chest in August 2017 identified resolution of the peripheral lung lesions, challenging the initial presumed diagnosis of malignancy.

**Figure 1:**
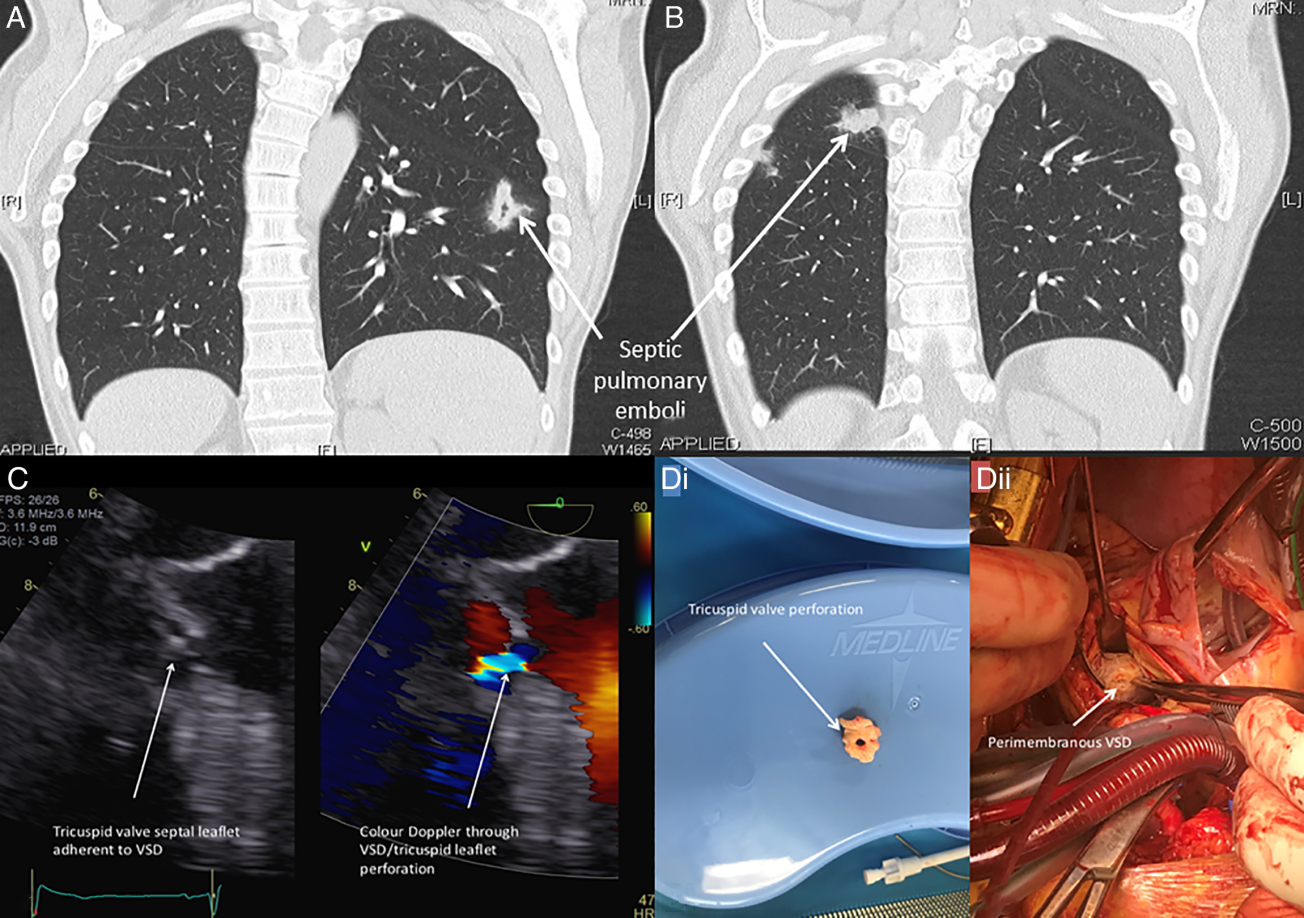
(A) Coronal computed tomography (CT) chest (lung window) showing abscess in left lung. (B) Coronal CT chest (lung window) showing abscess in right lung. (C) Transoesophageal echocardiogram short axis showing perimembranous ventricular septal defect (VSD) between 6 pm and 9 pm. (D) (i) Surgical specimen showing tricuspid valve septal leaflet perforation; (ii) operative field looking into left ventricle showing the perimembranous VSD under the aortic valve.

A further CT chest was organized in November 2017, and on this occasion there was recurrence of lung lesions in new areas, suggestive of septic emboli with internal cavitation (Fig. 1A, B). At this time that patient complained of sweats and lethargy, and was consequentially hospitalized for further investigation of his relapsing remitting lung lesions.

On admission, four blood cultures all identified *Streptococcus mutans*. A transthoracic and transoesophageal echocardiogram was completed, which identified a perimembranous VSD but no endocardial vegetation (Fig. 1C). A CT pulmonary angiogram was performed to exclude a pulmonary embolus and to identify an appropriate lung lesion to biopsy.

A thoracoscopic lung biopsy was completed, which isolated *S. mutans* from the necrotizing lung abscesses. Furthermore, the patient complained of lower back pain, which was investigated with spinal magnetic resonance imaging and identified early L3‐4 osteomyelitis without a complicating epidural abscess. As the patient was not haemodynamically compromised during admission, there was no need for urgent surgical repair of the VSD. The decision was made to treat the VSD‐related IE medically, followed by an elective VSD repair following a completed course of antibiotics. Initial medical treatment included six weeks of intravenous benzylpenicillin and ceftriaxone, followed by three months of oral amoxicillin.

The patient represented in February 2018 with recurrent sepsis without bacteraemia while on oral amoxicillin as a step down for *S. mutans* endocarditis. At this time, transoesophageal echocardiogram demonstrated the known VSD, as well as thickening of the adjacent right ventricle myocardium. Antibiotics were re‐escalated to intravenous benzylpenicillin and ceftriaxone for four weeks followed by VSD and tricuspid valve repair.

Intra‐operatively, the VSD was debrided and closed with a bovine pericardial patch. The adjacent tricuspid valve was excised en‐block and reconstructed using a sliding annuloplasty repair. Histopathology showed paucicellular fibrous tissue without evidence of active endocarditis, no organisms were cultured. Post‐operatively, the patient received intravenous benzylpenicillin and ceftriaxone for two weeks followed by oral amoxicillin for three months. He made a full recovery.

## Discussion

To our knowledge, this is the first reported case of *S. mutans* VSD‐related endocarditis presenting with recurrent and migratory lung nodules associated with possible osteomyelitis. Despite the patient being “high‐risk” for IE due to his VSD, the self‐limiting recurrent presentations, in association with his spontaneously resolving migratory lung lesions, delayed the diagnosis of septic pulmonary emboli and consequentially the diagnosis of IE 3. While it is possible the patient had recurrent IE from the time of his first presentation in 2016, it is unclear whether his presentation was related to a “low‐risk” dental procedure, or another non‐procedure bacteraemia.

The efficacy of anti‐microbial prophylaxis for IE in patients undergoing dental procedures is unknown 4. Antibiotic guidelines differ worldwide and prophylaxis is thought to prevent few cases of IE. For this reason, there has been a shift away from such practices and towards improved dental care 4. While VSD is considered low risk for bacterial endocarditis, our case highlights that VSD infection is possible and to consider this in patients with compatible symptoms.

We have reported a case of VSD‐related IE complicated by septic pulmonary emboli and possible osteomyelitis secondary to *S. mutans*. The patient made an uneventful recovery following antibiotic therapy and surgical intervention. Clinicians must have a high index of suspicion of IE in patients with structural heart disease presenting with recurrent fevers.

### Disclosure Statement

Appropriate written informed consent was obtained for publication of this case report and accompanying images.
